# Exposure to Food Marketing via Social Media and Obesity among University Students in Saudi Arabia

**DOI:** 10.3390/ijerph19105851

**Published:** 2022-05-11

**Authors:** Najlaa M. Aljefree, Ghada Talat Alhothali

**Affiliations:** 1 Food and Nutrition Department, Faculty of Human Sciences and Design, King Abdulaziz University, Building 43, Room 237, Level 2, Jeddah 3270, Saudi Arabia; 2 Marketing Department, College of Business, University of Jeddah, Building 17, Level 4, Room 4036, Jeddah 3795, Saudi Arabia; galhothali@uj.edu.sa

**Keywords:** obesity, food marketing exposure, social media platforms, food ads, unhealthy food, young adults

## Abstract

This study investigated the associations between obesity and unhealthy food/drink intake with both the frequency of social media platform usage and food/drink marketing exposure on social media. Data were obtained from 316 university students aged 18–29 years at two universities in Jeddah, Saudi Arabia. These participants completed online questionnaires with sections on demographics, anthropometric measurements, social media platform usage, food marketing exposure via social media, and unhealthy food consumption. All of the participants, 20.3% and 13.6% were overweight and obese, respectively. Snapchat was the most popular application (85.8%), followed by Instagram (75%), YouTube (61%), Twitter (51%), and TikTok (50%). The obese participants were more likely to purchase foods/drinks after watching relevant social media advertisements than their non-obese counterparts (*p* < 0.04). Moreover, those who purchased foods/drinks more frequently after watching such advertisements consumed higher amounts of potato chips (*p* < 0.01) and fast foods (*p* < 0.03). Finally, those who used Snapchat, TikTok, and Instagram tended to have higher consumption rates for potato chips (*p* < 0.02), fast foods (*p* < 0.01), sweets (*p* < 0.02), and sugary drinks (*p* < 0.04). Public health policymakers in Saudi Arabia should consider regulating unhealthy food and drink advertisements on social media platforms, especially those targeted at younger generations.

## 1. Introduction

Obesity is a serious public health concern that has attracted substantial attention from scholars, governments, and policymakers [1,2]. It is associated with a range of chronic illnesses, including diabetes, high cholesterol, hypertension, and heart disease [3,4,5], and has also been linked to higher rates of unemployment and reduced productivity, thereby impacting economic and societal conditions [6]. In turn, obesity diminishes the quality of life, with particularly negative impacts on the overall lifespan and general well-being [7,8]. In light of these issues, various studies have examined the prevalence of both overweightness and obesity, and their associations with sociodemographic characteristics in different nations, including the United States [9,10], the United Kingdom [11], and Australia [12]. Many studies have also focused on the Middle East and Gulf region [5,13], where there are increasing concerns over the prevalence of obesity and various contributing factors [14]. Whilst overweightness and obesity are, in general, becoming increasingly common, there are different trends between world regions [6]. This makes it important to explore regional variations and their causes, especially in order to ensure that any interventional efforts are properly tailored to their target populations [6].

Obesity is becoming highly prevalent among young adults in Saudi Arabia, emphasizing the need for early prevention measures [15]. Looking at the World Obesity Organization’s recommendations for treatment and management in the Gulf and Lebanon [16], no Gulf country is likely to meet the World Health Organization’s (WHO) goals for the reduction of childhood obesity by 2025. As such, governments and public health policymakers are actively seeking ways to address this problem. In Saudi Arabia, health promotion and disease prevention have been prioritized through the National Transformation Plan and Vision 2030, which aims to support public health and wellbeing [17]. Meanwhile, preventive policies include sugar taxes and caloric labelling requirements for meals and other consumable products. Indeed, a holistic approach is necessary. In addition to the well-known genetic, nutritional, economic, psychological, and pharmacological influences on obesity, there are other contributing factors [14]. These two major causes of the obesity pandemic (known as the “big two”) include inadequate physical exercise and the marketing of unhealthy foods and drinks [14,18]. 

Each year, food, beverage, and restaurant companies in the United States spend around $14 billion on advertising and promotional activities [19]. In fact, because the usage of social media is continuing to increase among different age groups globally, but more specifically among young generations [20], evidence shows that spending on social media advertising might reach more than USD 84 billion, which was the amount spent during 2019 [20]. Moreover, according to a content analysis of food advertisements on Arab television, several of the advertisements featured items that were high in calories, saturated fat, salt, and added sugar [21]. In the past, television advertising commonly targeted children and teenagers in the promotion of unhealthy foods, cereals, candies, and other sugar-added products [22]. Today, time spent on social media sites like Facebook, Twitter, and Instagram via mobile devices has increased, as a result of a decrease in the amount of time spent watching television, among young people. Social media commercials, company-generated content, branded games and apps, and paid promotions from bloggers and influencers are various ways in which food companies are responding to this marketing trend for younger consumers. In today’s world, food and beverage companies can reach kids anywhere and anytime through marketing trends disguised as amusement [19]. The problem is escalating, as the vast majority of the most extensively used social media sites do not impose full regulations on the advertising of unhealthy foods. As a result of the lack of comprehensive government regulation of the marketing of unhealthy foods on social media platforms, children and adolescents are in danger of being exposed to unhealthy food marketing on these platforms [20]. Hence, children and young adults are now bombarded with food promotions on social media platforms, including YouTube, Snapchat, and Instagram [23]. In particular, food advertising on television has been linked to an increase in childhood and adolescent obesity in previous research [21,24]; this study therefore hypothesizes that food advertising on social media would have the same effect on Saudi Arabia’s young population. In this context, reports from the General Authority of Statistics show that 98.43% of Saudi Arabia’s young population uses social media [23,25], highlighting the need to investigate how social media platform usage and food marketing exposure influence food consumption patterns and weight gain among the nation’s younger populations. Therefore, this study aims to examine the prevalence of obesity and its association with both the frequency of social media platform usage and food/drink marketing exposure via social media, with an additional focus on any relationships with unhealthy food intake.

## 2. Materials and Methods

### 2.1. Study Participants

A cross-sectional study was conducted among 316 public university students admitted to King Abdulaziz University and Jeddah University in Jeddah, Saudi Arabia, from August to December 2021. The total population of students in 2020 was 32,034 at King Abdulaziz University and 7576 at Jeddah University. Hence, the sample size required to achieve adequate statistical power was 269 based on a 90% confidence level [26]. Ethical approval for the study was obtained from the University of Jeddah Bioethics Committee of Scientific and Medical Research (UJ-REC-019).

### 2.2. Study Questionnaire

We gathered data through online questionnaires that were emailed to the students through the official systems at both King Abdulaziz University and the University of Jeddah. Therein, students were invited to participate in this research, contingent on providing their consent. They were also provided explanations of the study’s aims, a short introduction, and assurances of both their anonymity and the voluntary nature of participation. The questionnaire was adapted from prior studies [27,28] in which the English version of the questionnaire was initially used. After that, the questionnaire was translated from English into Arabic, and then back again. In social science research, back translation is frequently used to guarantee that translations are accurate [29]. In order to properly address concerns about comprehension and meaning for the target respondents, back translation cannot be used solely. For this reason, it is extremely important to conduct the pre-testing of the questionnaire amongst members of the potential participants [30]. In this study, as an added measure of validity, two food and nutrition experts, as well as a number of marketing scholars, were given a copy of the survey to look over and comment on. Based on face validity, it appeared to be an easy and understandable questionnaire for potential respondents to complete. Then, pilot testing on a group of students (*n* = 15) was carried out in order to ensure that no questions were misunderstood, perceived incorrectly, or difficult to understand. There were four sections in the final questionnaire, on demographic characteristics/anthropometric measurements, social media platform usage, food marketing exposure via social media, and unhealthy food consumption. 

### 2.3. Demographic Characteristics and Anthropometric Measurements

The questionnaire began with demographic items, including gender, age, marital status, academic year, family income, height, and weight; the latter two were used to calculate body mass index (BMI) values for each participant. According to the WHO criteria [31], overweightness was established provided that the BMI values were between 25 and 29.9 kg/m^2^, while obesity was established provided that the values were ≥30 kg/m^2^.

### 2.4. Social Media Usage and Food Marketing Exposure via Social Media

The first set of items targeted the frequency of social media platform usage, including Snapchat, Facebook, Instagram, YouTube, Twitter, and TikTok, with response options including daily, weekly, monthly, and never. The participants were also presented with the following three items on exposure to food/drink marketing via social media platforms: (1) their frequency of viewing food/drink advertisements on social media platforms during the previous month, with response options including 3–4 times/day, once/day, 2 times/week, 2 times or less per month, and rarely; (2) whether they had purchased foods/drinks after exposure to related advertising on social media during the previous month, with response options including yes and no; and (3) how many times they had purchased foods/drinks after exposure to such advertisements during the last month, with response options including ≥2 times and one time or less.

### 2.5. Unhealthy Food Consumption

The participants were asked how frequently they had consumed unhealthy food/drink items during the previous month, including fast foods (e.g., burgers, pizza, McDonalds, shawarma, fried chicken), potato chips (including salty snacks and corn chips), sweets (e.g., chocolate, cakes, donuts, ice cream), and sugary drinks (soft drinks, sports drinks). In each case, the response options included ≥2 times/day and rarely or never, which were respectively dichotomized as ≥2 times/week and <2 times/week.

## 3. Statistical Analysis

We used the IBM Statistical Package for Social Science (SPSS; version 28) to conduct the data analyses, including descriptive analyses with frequencies and percentages. In order to assess any differences between obese and non-obese participants, we conducted a chi-square test to compare their demographic characteristics, frequency of social media platform usage, and exposure to food/drink marketing via social media platforms. We also conducted a chi-square test to compare exposure to food/drink marketing via social media platforms between participants with high and low unhealthy food/drink intake. Due to the small sample size, we considered overweight and obese participants as one group, i.e., the “obese group”. We conducted separate logistic regression analyses to examine the associations between unhealthy food intake and the frequency of social media platform usage. As only a few participants answered “never” to all social media platform usage except for Facebook, we combined those who answered “monthly” and “never” into one group for the logistic regression. In similar regard, for the frequency of Facebook usage, we combined the participants who answered “daily” and “weekly” into one group. We adjusted all of the models for age, sex, nationality, marital status, academic year, monthly household income, and BMI. Statistical significance was set at *p* < 0.05.

## 4. Results

Table 1 lists the demographic characteristics reported by the study participants according to their obesity status. There was a total of 316 participants, the majority of whom were female (71.5%), Saudi nationals (79.8%), between 18 and 21 years of age (74.5%), and of single marital status (94.6%). Compared to the non-obese group, the obese group included a large number of female (*p* < 0.001) participants in their second and fourth years at university (*p* < 0.001). Of all of the participants, 20.3% and 13.6% were overweight and obese, respectively.

**Figure 1:**
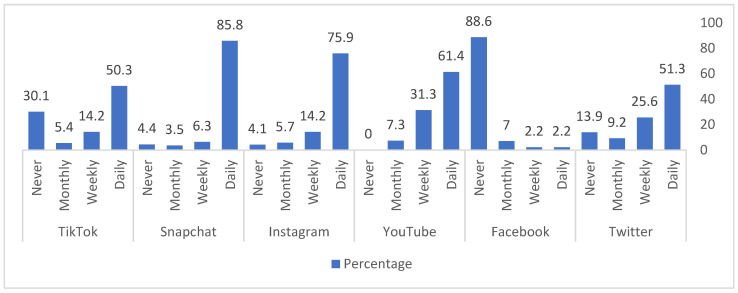
The percentage of the use of social media platforms among the study participants.

Table 2 lists the frequency of social media platform usage according to obesity status. Snapchat (85.8%), Instagram (75.9%), YouTube (61.4%), Twitter (51.3%), and TikTok (50.3%) were most frequently used on a daily basis. Facebook was the least commonly used (88.6% reported no usage) (Figure 1). There was no significant association between the frequency of social media platform usage and obesity. 

Table 3 lists the exposure to food/drink marketing via social media platforms according to obesity status. Over half of all of the participants reported exposure rates of three to four times per day during the previous month (54%), with lesser percentages reporting one time per day (21.5%) and two times per week (13.6%). Moreover, nearly half of the participants said they had purchased food and drinks after watching related advertisements on social media platforms (46.8%). Compared to participants in the non-obese group, those in the obese group had more often purchased food and drinks two or more times per week during the previous month after watching such advertisements (*p* < 0.04).

Table 4 shows the association between exposure to food marketing via social media platforms and unhealthy food/drink intake. The participants who purchased food and drinks because they had watched related advertisements on social media consumed higher amounts of potato chips (*p* < 0.04) than the participants who did not. Compared to the participants who only purchased food and drinks one time or less per week after watching related advertisements on social media, those who purchased food and drinks two times or more per week consumed higher amounts of potato chips (*p* < 0.01) and fast foods (*p* < 0.03).

Table 5 shows the associations between unhealthy food intake and the frequency of social media platform usage. There was a non-significant association between the frequency of YouTube, Twitter, and Facebook usage and unhealthy food consumption (including potato chips, sweets, fast foods, and sugary drinks). Compared with the participants who used TikTok on a daily basis, less frequent users (never/monthly) were less likely to consume fast foods (OR: 0.45, 95% CI: 0.24–0.84, *p* < 0.01). In similar regard, compared to the participants who used Snapchat daily, less frequent users (never/monthly) were less likely to consume potato chips (OR: 0.29, 95% CI: 0.8–1.04, *p* < 0.05), sweets (OR: 0.3, 95% CI: 0.109–0.86, *p* < 0.02), and sugary drinks (OR: 0.38, 95% CI: 0.15–1.01, *p* < 0.05). Meanwhile, moderate Snapchat users (weekly) were less likely to consume potato chips (OR: 0.09, 95% CI: 0.01–0.76, *p* < 0.02) and sugary drinks (OR: 0.33, 95% CI: 0.11–0.98, *p* < 0.04). Finally, compared to participants who used Instagram on a daily basis, moderate users (weekly) were more likely to consume sugary drinks (OR: 1.9, 95% CI: 0.98–3.7, *p* < 0.05).

## 5. Discussion

This study examined the prevalence of obesity among university students, and how it was associated with both the frequency of social media platform usage and food/drink marketing exposure via social media. We also focused on the association between these factors and unhealthy food intake. There were some interesting findings. First, 20.3% and 13.6% of all participants were overweight and obese, respectively. The obese group had a large number of female participants in their second and fourth years at university. This former result is similar to the findings of previous studies showing a higher prevalence of obesity among female adolescents in Saudi Arabia [32]. As for exposure to food/drink marketing via social media platforms, the obese participants were more likely than their non-obese counterparts to purchase food and drinks because they had watched related advertisements. This supports previous findings demonstrating an association between unhealthy food consumption and exposure to food advertisements [27]. Thus, the primary hypothesis was confirmed. Additionally, the participants who purchased food and drinks more frequently (two or more times per week) after watching related advertisements on social media platforms consumed higher amounts of potato chips and fast foods. There was also a non-significant association between the frequency of social media platform usage and obesity. Looking at specific platforms, the participants who used Snapchat, TikTok, and Instagram more frequently consumed potato chips, fast foods, sweets, and sugary drinks.

Obesity has become a significant epidemic in developed and developing countries. In recent years, the prevalence of this condition greatly increased across all age groups, particularly in young adults [33,34,35]. Previous research has also shown that young adults are at high risk of developing overweightness and obesity, especially during their first years at university. In fact, studies have shown that university students gain around 3.5 kg of weight over this time, partly due to unhealthy eating behaviors [33]. In this study, around one-third of all participants were classified as either overweight or obese, which supports previous findings from Saudi Arabia. For example, Al-Rethaiaa et al. [34] reported that 21.8% and 15.7% of students who were studying at public university in Saudi Arabia were overweight and obese, respectively; the same problems exist among other public university students in developing countries, including Malaysia (15.9% vs. 5.2%) [35] and Egypt (36.9% vs. 12.5%) [36]. Surprisingly, Al Turki et al. [37] found even higher rates of overweightness and obesity among students who were studying in public university in Riyadh, at 31% and 23.3%, respectively. 

Social media platform usage is now popular across the globe, especially among young adults [23]. For example, around 73% of young adults in the United States have registered accounts with at least one platform [38]. Meanwhile, usage has substantially increased in Saudi Arabia, where rates are thought to be among the highest in the world [35]. In 2021, approximately 79% of the Saudi population (27.8 million) were considered active social media users, with three-quarters of these being young adults [39]. The State of Social Media in Saudi Arabia (published in 2012 and 2013) showed that the Kingdom had the highest national rankings in both YouTube video views (90 million) and Twitter usage [40]. In this study, Snapchat (85.8%), Instagram (75.9%), YouTube (61.4%), Twitter (51.3%), and TikTok (50.3%) were the most commonly used social media platforms. These results are consistent with reports from the United States, where YouTube (80%), Instagram (72%), Snapchat (69%), Facebook (51%), and Twitter (32%) were the most frequently used platforms among young adults [41]. Likewise, Facebook, Twitter, and YouTube were the premier social media platforms in the Arabic Gulf region as of 2014 [42]. It is noteworthy that this study’s results generally align with previous findings [41,42], except for those on Facebook, for which our participants reported very low usage; in fact, 88.6% of the participants reported that they never used Facebook. This may be due to declining rates. While Facebook was the most frequently used social media site in Saudi Arabia in 2011, it lost this position to Twitter in 2013 [42]. Moreover, a 2021 study conducted in Saudi Arabia showed that Snapchat, Twitter, Instagram, and YouTube were the most desired social media platforms [43], which is consistent with this study’s findings. 

Food and drink marketing on social media platforms has a powerful influence on eating habits among young populations [44]. Because young adults usually spend more time browsing the Internet, own personal smartphones, and are registered on more than one social media channel, they are heavily exposed to food and drink advertisements, especially in comparison to other forms of marketing [43,44]. In this study, the participants generally had high exposure to food and drink marketing via social media platforms, with about half reporting exposure three to four times per day, and one-quarter reporting once per day during the last month. We also found that close to half of all of the participants had directly purchased food and drinks because they saw related advertisements on social media. In this context, obese students showed a greater tendency than their non-obese counterparts. This supports Scully et al. (2012), who found that students who were exposed to digital food marketing were more likely to buy the products they had seen in those advertisements [28]. 

A study conducted in the United States reported that 68% of food marketing on social media platforms promoted unhealthy foods and sugary drinks [44]. This is a serious problem considering the evidence showing that young adults are highly influenced by unhealthy food advertisements on social media, especially as opposed to advertisements featuring healthy foods. Fleming-Milici and Harris [45] found that nearly half of their study population was involved with unhealthy foods and drinks on social media, including fast foods, sugary drinks, and snacks [45]. Similarly, one study found that Saudi adults who were exposed to food marketing on social media platforms sought unhealthy foods, including burgers, pizza, cookies, and chocolate; by contrast, healthy food items were less desired, including Greek yogurt, granola, and sugar-free juices [43]. Comparably, Rummo et al. [46] found that a high number of adolescents in the United States followed unhealthy food and drink brands on Twitter and Instagram, particularly those associated with fast foods and sugary drinks [46]; the authors concluded that this was likely to increase consumption, thereby increasing weight gain.

The current findings also highlight the link between exposure to food/drink marketing via social media platforms and unhealthy food consumption. Previously, Buchanan et al. [47] reported that this type of exposure was associated with increased energy drink consumption, while a study among Australian adolescents found that high exposure was positively associated with increased unhealthy food and drink intake [47]. Indeed, we found that participants who used Snapchat, TikTok, and Instagram were more likely to consume potato chips, fast foods, sweets, and sugary drinks. Thus, it provided evidence that social media food marketing has powerful impacts on individual eating behaviors and food choices, especially in younger demographics. In turn, this increases the risk of obesity and other non-communicable diseases. 

Overall, this study’s findings suggest that policymakers should regulate unhealthy food and drink advertisements on social media, especially those targeted at younger viewers. Given the influential impacts on consumption in this demographic, other marketers should use social media to promote healthy foods, drinks, and lifestyle habits. Meanwhile, companies that produce unhealthy foods and drinks should add healthy ingredients to their products or offer other product lines. Educational institutions should also reinforce the importance of healthy behaviors by conducting awareness campaigns that are targeted at younger generations, especially concerning the need for healthy food intake and the negative impacts that obesity has on health and wellbeing. Moreover, campaigns that foster the importance of adopting healthy lifestyles are essential, as they constitute an important objective outlined in the ambitious Saudi Vision 2030. This may require the implementation of healthy activities as part of school curricula, whilst also ensuring that university students are able to access healthy foods and drinks on campus. For example, local vending machines, cafés, and shops should provide a greater variety of healthy meal options and other food products. In addition, social media platforms should apply strict regulations for the advertising of unhealthy food and drinks for young people, and should utilize a comprehensive definition of the term “unhealthy food and drinks.” This will enhance public health and reduce the burden of chronic diseases in future generations. Indeed, a few social media platforms such as Facebook and Instagram have used rigorous policies on the advertising of weight loss products and tobacco use to individuals under 18 years of age [20]. However, the regulations on the advertising of unhealthy food and drinks on social media platforms were not inclusive and effective, as they did not reduce the exposure to unhealthy food/drinks marketing among young people.

This study also had some limitations that should be discussed. First, we collected cross-sectional data from students at only two public universities, which highlights the need for additional sampling that includes students who attend private universities. Second, we limited our examination to food marketing exposure via social media, but it is also important to assess the impacts of direct engagement with food and drink marketing in the social media context, especially with regard to the way in which this is connected with obesity. Similarly, studies should also investigate how marketing from social media influencers affects food consumption and purchasing behaviors in young adults using a qualitative approach. Furthermore, additional research is necessary in order to investigate whether the exposure of adults and children to food advertising via social media is connected with weight increase during Ramadan, and if so, whether this weight gain contributes to increased yearly overweightness and obesity [21]. Moreover, food advertising has been linked to a rise in young adult obesity; as such, it is worth looking into whether healthy food marketing plays a role. Third, we used self-reported height and weight to calculate the BMI. While there are known limitations to the use of self-reported data, it is worth noting that the same method is widely used in health research for economic purposes [48]. This is also an important method for collecting data under the constraints of the COVID-19 pandemic, as online questionnaires are often the preferred means. Finally, the participants may have been affected by recall bias, as they needed to remember their food/drink intake and social media usage rates over a one-month period. 

## 6. Conclusions

Weight gain has increased dramatically in recent decades, such that it is now a significant public health problem for current and future generations across the world. In this context, the prevalence of obesity has substantially increased across all age groups, especially among young adults. In order to combat this problem, several multidisciplinary studies have investigated obesity and its contributing factors, with the aim of finding the best methods for reducing its incidence in the general population. In turn, public policymakers and governments have responded by implementing relevant preventive and protective measures. Here, many researchers have found that food marketing is a major contributing factor. In fact, a large number of international studies have reported statistically significant links between exposure to food/drink marketing via social media and both unhealthy food consumption and weight gain, although there is only limited evidence on these relationships in Saudi Arabia. This study addressed this gap by targeting Saudi university students, who are among the most commonly affected demographics. In support of existing evidence, it was found that the obese participants were more likely than their non-obese counterparts to purchase foods and drinks after watching related advertisements on social media platforms. Moreover, the participants who purchased foods and drinks more frequently because of such advertisements consumed higher amounts of potato chips and fast foods, while those who used Snapchat, TikTok, and Instagram more frequently consumed high amounts of potato chips, fast foods, sweets, and sugary drinks. Looking at specific platforms of interest, we found that Snapchat was the most popular, followed by Instagram, YouTube, Twitter, and TikTok.

These findings provide a warning regarding public health issues related to obesity in Saudi Arabia, to policymakers and government organizations, including the Saudi Food and Drug authority. In this regard, policymakers should stringently regulate unhealthy food and drink advertisements on social media platforms, especially considering the evidence that three-quarters of social media users in the Kingdom are young adults. In general, there must be higher awareness among the population of how obesity impacts health, as well as its associations with exposure to food and drink marketing via social media, especially in younger demographics. The importance of healthy behaviors should be reinforced by educational institutions, for example, through the use of awareness campaigns that target young people. These campaigns should emphasize the importance of a healthy diet and the negative effects that obesity has on one’s health and wellbeing.

## Figures and Tables

**Table 1:** Demographic characteristics of the study participants according to their obesity status.

Demographic Characteristics	*n* (%)	Obesity		*p* Value
		Non-Obese Participants	Obese Participants	
Gender				**<0.001**
Male	90 (28.5)	38 (18.7)	50 (46.7)	
Female	226 (71.5)	165 (81.3)	57 (53.3)	
Age group (years)				0.202
18–21	235 (74.5)	153 (75.4)	76 (71)	
22–25	77 (24.5)	49 (24.1)	28 (26.2)	
26–29	4 (1.3)	1 (0.5)	3 (2.8)	
Nationality				0.094
Saudi	309 (79.8)	201 (99)	103 (96.3)	
Non-Saudi	7 (2.2)	2 (1)	4 (3.7)	
Marital status				0.563
Single	299 (94.6)	193 (95.1)	100 (93.5)	
Married	16 (5.1)	9 (4.4)	7 (6.5)	
Divorced/widowed	1 (0.3)	1 (0.5)	0	
Academic years				**<0.001**
First	84 (26.6)	64 (31.5)	17 (15.9)	
Second	65 (20.6)	30 (14.8)	34 (31.8)	
Third	64 (20.3)	47 (23.2)	15 (14)	
Fourth	70 (22.2)	44 (21.7)	26 (24.3)	
Fifth	19 (6)	11 (5.4)	8 (7.5)	
Sixth	14 (4.4)	7 (3.4)	7 (6.5)	
Monthly household income (SAR */monthly):				0.403
<3000	57 (18)	33 (16.3)	23 (21.5)	
3000–7000	61 (19.3)	36 (17.7)	24 (22.4)	
7000–12,000	76 (24.1)	53 (26.1)	22 (20.6)	
12,000–20,000	63 (19.9)	44 (21.7)	17 (15.9)	
>20,000	59 (18.7)	37 (18.2)	21 (19.6)	

*p*-values based on an X^2^ test; bold indicates significance; *p* < 0.05; * Saudi Riyal (SAR 1 = USD 0.27).

**Table 2:** Frequency of social media platform usage among the study participants according to their obesity status.

How Often Do You Use the Following?	*n* (%)	Obesity		*p* Value
		Non-Obese Participants	Obese Participants	
**Twitter:**				0.502
Daily	162 (51.3)	109 (53.7)	50 (46.7)	
Weekly	81 (25.6)	51 (25.1)	27 (25.2)	
Monthly	29 (9.2)	16 (7.9)	13 (12.1)	
Never	44 (13.9)	27 (13.3)	17 (15.9)	
**Facebook:**				0.236
Daily	7 (2.2)	3 (1.5)	4 (3.7)	
Weekly	7 (2.2)	3 (1.5)	4 (3.7)	
Monthly	22 (7)	16 (7.9)	5 (4.7)	
Never	280 (88.6)	181 (89.2)	94 (87.9)	
**YouTube:**				0.873
Daily	194 (61.4)	123 (60.6)	68 (63.6)	
Weekly	99 (31.3)	65 (32)	32 (29.9)	
Monthly	23 (7.3)	15 (7.4)	7 (6.5)	
Never	0	0	0	
**Instagram:**				0.897
Daily	240 (75.9)	156 (67.8)	78 (72.9)	
Weekly	45 (14.2)	28 (13.8)	17 (15.9)	
Monthly	18 (5.7)	11 (5.4)	7 (6.5)	
Never	13 (4.1)	8 (3.9)	5 (4.7)	
**Snapchat:**				0.922
Daily	271 (85.8)	172 (84.7)	93 (86.9)	
Weekly	20 (6.3)	14 (6.9)	6 (5.6)	
Monthly	11 (3.5)	8 (3.9)	3 (2.8)	
Never	14 (4.4)	9 (4.4)	5 (4.7)	
**TikTok:**				0.545
Daily	159 (50.3)	107 (52.7)	49 (45.8)	
Weekly	45 (14.2)	28 (13.8)	17 (15.9)	
Monthly	17 (5.4)	9 (4.4)	8 (7.5)	
Never	95 (30.1)	59 (29.1)	33 (30.8)	

*p*-values based on a X^2^ test.

**Table 3:** Exposure to food marketing via social media platforms among the study participants according to their obesity status.

Exposure to Food Marketing	*n* (%)	Obesity		*p* Value
		Non-Obese Participants	Participants with Obesity	
How often did you watch food and drink ads on social media websites during the last month?				
3–4 times/day	171 (54.1)	112 (55.2)	55 (51.4)	
Once/day	68 (21.5)	40 (19.7)	28 (26.2)	
2 times/week	43 (13.6)	27 (13.3)	15 (14)	
≤2 times/month	6 (1.9)	3 (1.5)	3 (2.8)	
Rarely	28 (8.9)	21 (10.3)	6 (5.6)	0.408
Did you purchase any foods and/or drinks because you saw ads for them on social media websites during the last month?				
Yes	148 (46.8)	90 (44.3)	55 (51.4)	
No	168 (53.2)	113 (55.7)	52 (48.6)	0.143
How many times have you purchased foods and/or drinks because you saw ads for them on social media websites?				
≥2 times	157 (49.7)	91 (44.8)	61 (57)	
One time or less	159 (50.3)	112 (55.2)	46 (43)	**0.041**

*p*-values based on a X^2^ test; bold indicates significance; *p* < 0.05.

**Table 4:** Exposure to food marketing via social media websites and its association with unhealthy food intake among the study participants.

Unhealthy Food	How Many Times Did You Watch Food and/or Drink Ads on Social Media Websites during the Last Month?				Did You Purchase Any Foods and/or Drinks Because You Saw Ads for Them on Social Media Websites during Last Month?			How Many Times Have You Purchased Foods and/or Drinks Because You Saw Ads for Them on Social Media Websites?		*p*
	Daily *n* (%)	Weekly *n* (%)	≤2 Times/ Month or Rarely *n* (%)	*p*	Yes *n* (%)	No *n* (%)	*p*	≥2 Times *n* (%)	One Time or Less *n* (%)	
**Potato chips**										
**<2 times/week**	178 (74.5)	33 (76.6)	26 (76.5)	0.93	104 (70.3)	133 (79.2)	**0.04**	108 (68.8)	129 (81.1)	**0.01**
**≥2 times/week**	61 (25.5)	10 (23.3)	8 (23.5)		44 (29.7)	35 (20.8)		49 (31.2)	30 (18.9)	
**Sweets**										
**<2 times/week**	136 (56.9)	29 (67.4)	19 (55.9)	0.41	91 (61.5)	93 (55.4)	0.162	48 (53.5)	100 (62.9)	0.09
**≥2 times/week**	103 (43.1)	14 (32.6)	15 (44.1)		57 (38.5)	75 (44.6)		73 (46.5)	59 (37.1)	
**Fast foods**										
**<2 times/week**	165 (69)	30 (69.8)	24 (70.6)	0.98	95 (64.2)	124 (73.8)	0.06	100 (63.7)	119 (74.8)	**0.03**
**≥2 times/week**	74 (31)	13 (30.2)	10 (29.4)		53 (35.8)	44 (26.2)		57 (36.3)	40 (25.2)	
**Sugary drinks**										
**<2 times/week**	133 (55.6)	21 (48.8)	20 (58.8)	0.63	83 (56.1)	91 (54.2)	0.73	83 (52.9)	91 (57.2)	0.43
**≥2 times/week**	106 (44.4)	22 (51.2)	14 (41.2)		65 (43.9)	77 (45.8)		74 (47.1)	68 (42.8)	

*p*-values based on a X^2^-test; bold indicates significance; *p* < 0.05.

**Table 5:** Associations between the intake of unhealthy food items and the frequency of using social media platforms.

The Frequency of Using Social Media Sites	Unhealthy Eating Behaviors							
	Potato Chips *		Sweets *		Fast Foods *		Sugary Drinks *	
	OR	(95% CI) *p*	OR	(95% CI) *p*	OR	(95% CI) *p*	OR	(95% CI) *p*
**Twitter:**								
Daily	1		1		1		1	
Weekly	1.07	(0.57–2) 0.84	0.8	(0.45–1.4) 0.45	0.74	(0.39–1.4) 0.37	0.97	(0.55–1.7) 0.94
Monthly/Never	0.88	(0.44–1.7) 0.73	0.92	(0.51–1.6) 0.79	0.74	(0.39–1.4) 0.35	0.89	(0.5–1.6) 0.72
**YouTube:**								
Daily	1		1		1		1	
Weekly	0.81	(0.44–1.5) 0.52	1.2	(0.73–2) 0.42	0.87	(0.48–1.5) 0.64	0.88	(0.51–1.4) 0.63
Monthly/Never	1.8	(0.71–4.8) 0.207	0.96	(0.38–2.4) 0.94	1.3	(0.5–3.7) 0.52	1.3	(0.41–2) 0.49
**Instagram:**								
Daily	1		1		1		1	
Weekly	0.73	(0.31–1.6) 0.45	1.07	(0.55–2) 0.83	1.1	(0.57–2.3) 0.67	1.9	(0.98–3.7) 0.05
Monthly/Never	0.903	(0.35–2.2) 0.82	0.48	(0.2–1.1) 0.09	0.91	(0.38–2.1) 0.83	0.91	(0.2–1.6) 0.81
**Snapchat:**								
Daily	1		1		1		1	
Weekly	0.09	(0.01–0.76) 0.02	0.96	(0.37–2.4) 0.93	0.29	(0.08–1) 0.06	0.33	(0.11–0.98) 0.04
Monthly/Never	0.29	(0.8–1.04) 0.05	0.3	(0.109–0.86) 0.02	0.6	(0.2–1.6) 0.32	0.38	(0.15–1.01) 0.05
**TikTok:**								
Daily	1		1		**1**		1	
Weekly	0.91	(0.41–2) 0.83	1.05	(0.52–2) 0.89	**0.94**	**(0.45–1.9) 0.88**	1.3	(0.70–2.7) 0.34
Monthly/Never	0.78	(0.42–1.4) 0.45	0.74	(0.43–1.2) 0.27	**0.45**	**(0.24–0.84) 0.01**	0.96	(0.56–1.6) 0.903
**Facebook:**								
Never	1		1		1		1	
Monthly	1.6	(0.46–5.8) 0.44	0.25	(0.05–1.2) 0.08	0.41	(0.1–1.6) 0.21	0.75	(0.24–2.3) 0.63
Daily/Weekly	0.88	(0.29–2.6) 0.83	1.03	(0.4–2.6) 0.94	2.06	(0.77–5.5) 0.14	1.9	(0.73–4.9) 0.18

Bold indicates significance *p* < 0.05; OR = adjusted odds ratio; CI = confidence intervals; * logistic regression models after adjusting for age, sex, nationality, marital status, academic year, monthly household income, and BMI.

## Data Availability

None of the datasets generated and/or analyzed in this study are publicly available because the authors require them for further publication. However, the data are available from the corresponding author upon request. Signed online consent forms were obtained from all of the participants prior to data collection.
